# Solvent-mediated conductance increase of dodecanethiol-stabilized gold nanoparticle monolayers

**DOI:** 10.3762/bjnano.7.196

**Published:** 2016-12-23

**Authors:** Patrick A Reissner, Jean-Nicolas Tisserant, Antoni Sánchez-Ferrer, Raffaele Mezzenga, Andreas Stemmer

**Affiliations:** 1 ETH Zürich, Nanotechnology Group, Säumerstrasse 4, CH-8803 Rüschlikon, Switzerland; 2 ETH Zürich, Laboratory of Food and Soft Materials, Schmelzbergstrasse 9, CH-8092 Zürich, Switzerland

**Keywords:** molecular electronics, molecular exchange, percolation networks, Self-assembly

## Abstract

Gold nanoparticle monolayers provide convenient templates to study charge transport in organic molecules beyond single junction techniques. Conductance is reported to increase by several orders of magnitude following immersion of alkanethiol-stabilized gold nanoparticle monolayers in a solution containing conjugated thiol-functionalized molecules. Typically, this observation is attributed to molecular exchange. Less attention has been paid to the role of the solvent alone. Here, we report on an increase in conductance of dodecanethiol-stabilized gold nanoparticle monolayers on Si/SiO_2_ by an average factor of 36 and 22 after immersion in pure ethanol (EtOH) and tetrahydrofuran (THF), respectively. Analysis by scanning electron microscopy (SEM) and small-angle X-ray scattering (SAXS) reveals a solvent-induced decrease in lattice constant of close-packed monolayers. We compare the conductance of the monolayer after molecular exchange with two different oligophenylenes to shed light on the respective contribution of the solvent-induced structural change and the molecular exchange itself on the conductance increase.

## Introduction

Ordered gold nanoparticle monolayers are increasingly applied as templates for molecular resistor networks [1–8]. Gold nanoparticles serve as conducting nodes and different molecules can bind to the gold nanoparticle using anchoring groups such as thiols or amines [3,9–10]. The conductance between neighboring nanoparticles depends on a multitude of factors including the conductivity of surrounding molecules, the type of bond between molecules and nanoparticles and the interparticle distance [3,5–6]. The conductivity of the entire network further depends on the percolation of charge carriers [11–12]. Initially, gold nanoparticles are typically stabilized by alkanethiol ligands, which are poor conductors. As reported, the conductivity of nanoparticle networks can be increased by immersing the substrate with the nanoparticle monolayer in a solution containing conjugated molecules [3,5–7,13]. So far, the observed overall change in conductivity has been interpreted in terms of a molecular exchange and little or no attention has been paid to the role of the solvent itself on this effect.

In the following, we demonstrate that the solvent alone can induce a structural transition responsible for a large portion of the observed increase in conductivity of micro-contact printed self-assembled gold nanoparticle monolayers.

## Results and Discussion

**Figure 1:**
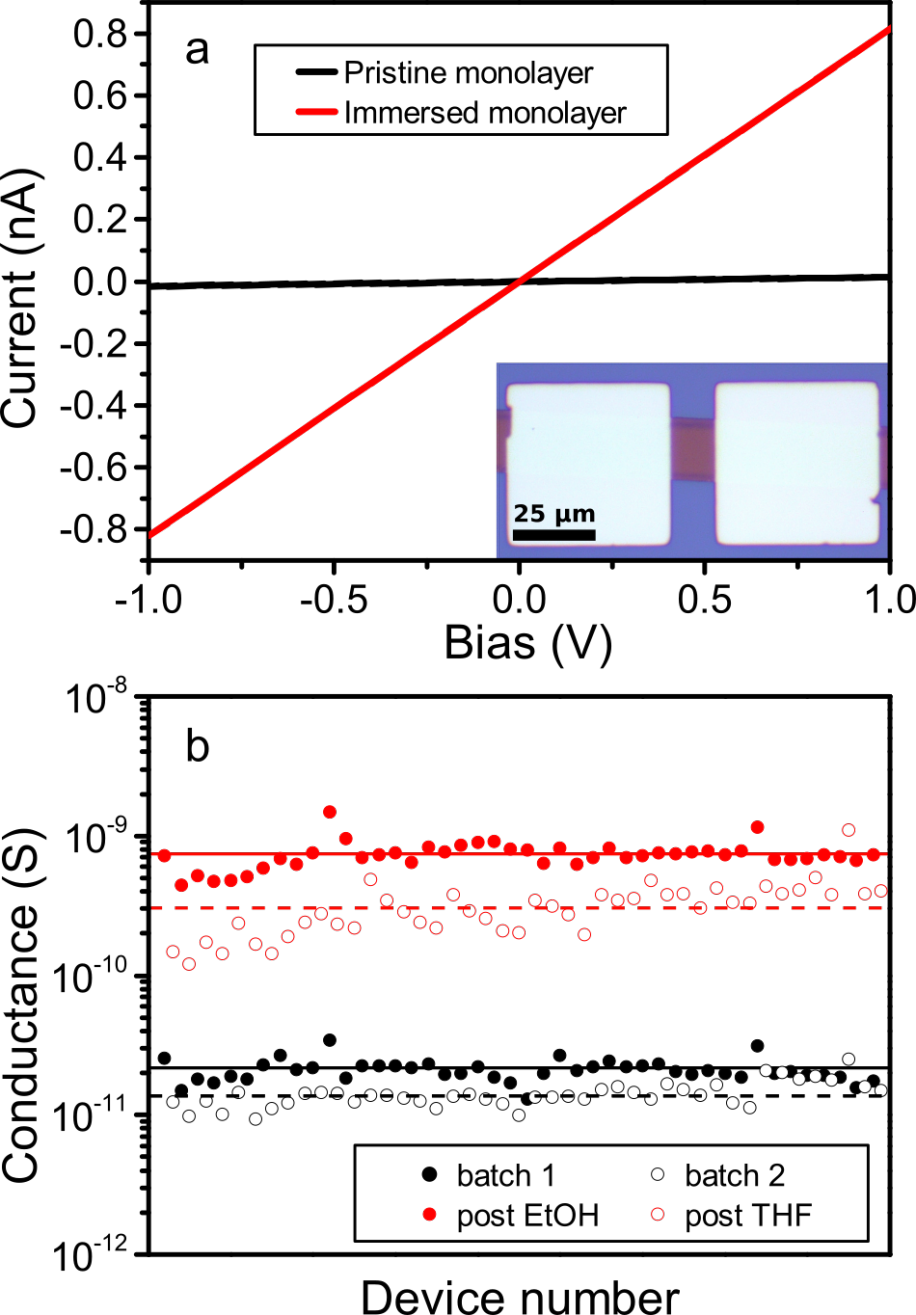
(a) Current–voltage relation of 20 µm × 10 µm gold nanoparticle monolayer before (black) and after (red) immersion in EtOH. The inset shows an optical image of a representative device. (b) Conductance values measured for 44 devices and their mean values of two different batches before (black) and after (red) solvent immersion. Full and empty symbols represent measurements of devices of batch 1 and 2, respectively.

Gold nanoparticles with an average diameter of 10.6 nm measured by small-angle X-ray scattering (SAXS) were synthesized [14], functionalized by 1-dodecanethiol [15], assembled to form a monolayer [16], and deposited onto an Si/SiO_2_ substrate using a patterned poly(dimethylsiloxane) (PDMS) stamp [17]. A detailed description of all steps is provided in the methods section. The resulting pattern of the nanoparticle monolayer consists of 20 µm wide lines spaced 100 µm apart. An optical image of the resulting electrical devices used in this study is shown in the inset of Figure 1a. The red nanoparticle array was electrically contacted by a pair of gold electrodes separated 10 µm apart.

The conductance of such devices was measured by acquiring *I*–*V* curves before and after immersing them in pure solvents. All devices exhibit a linear current–voltage response before and after solvent immersion as shown in Figure 1a for a randomly picked device. The differential conductance value of each device is plotted in Figure 1b. Black data points show the conductance of the pristine devices. The average conductance of devices of batch 1 and batch 2 before immersion amounts to 2.2 × 10^−11^ S and 1.4 × 10^−11^ S, respectively. We immersed devices of batch 1 in ethanol (EtOH) and batch 2 in tetrahydrofuran (THF) for 20 h. Subsequent measurements of the dried devices (red data points in Figure 1) showed an average increase in conductance by a factor of 36 for EtOH immersion and 22 for THF immersion. Conductance changes of alkanethiol-stabilized gold nanoparticle multilayers were also reported upon exposure to solvent vapor [18–19]. However, these changes were reversible in the absence of the vapor. In our case, the change in conductance is permanent and accompanied by structural changes, as we will show in the following.

**Figure 2:**
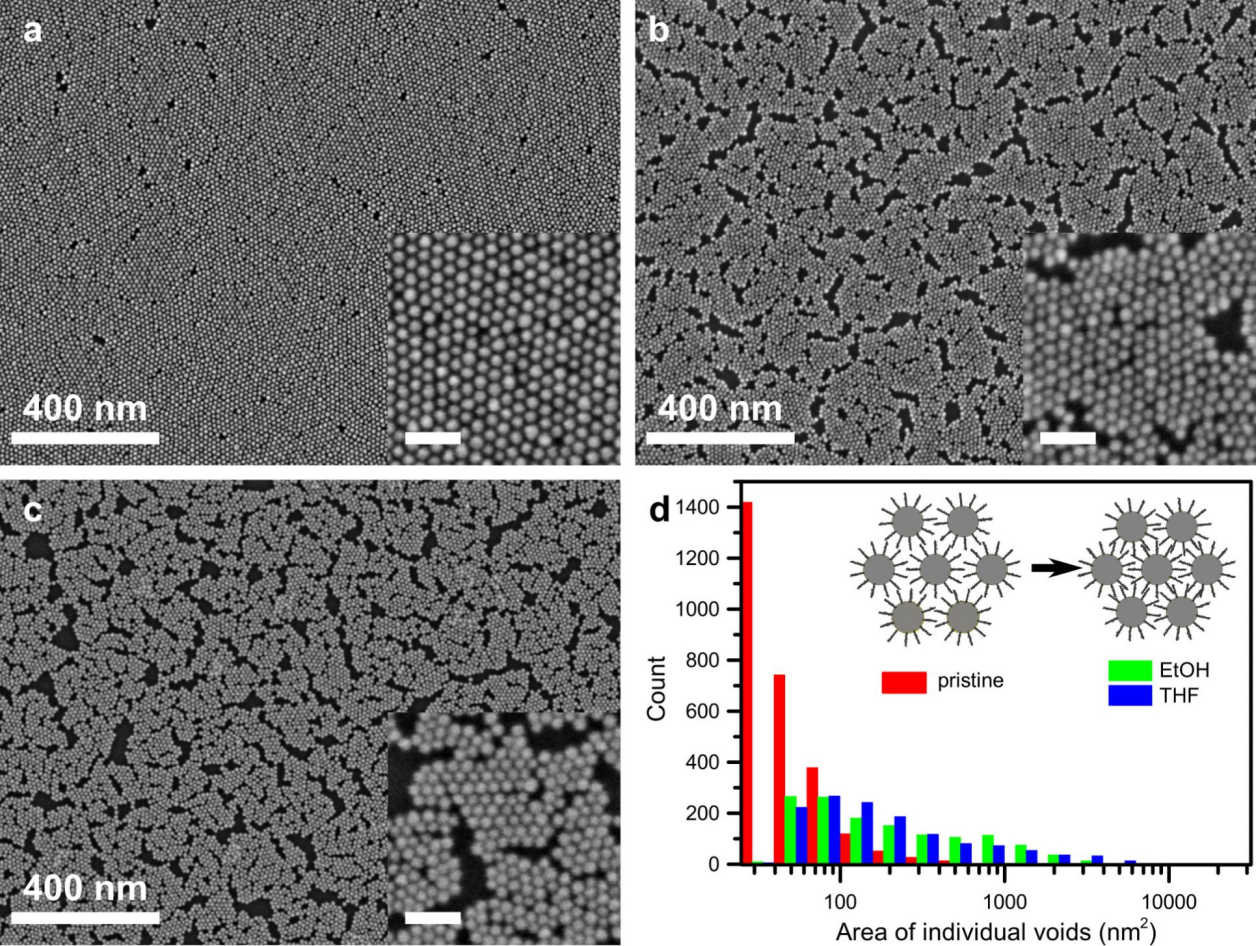
SEM images of (a) a pristine gold nanoparticle monolayer, (b) after EtOH immersion and (c) after THF immersion. Scale bar inset is 50 nm. (d) Histograms of the area of individual voids of the SEM images shown in (a)–(c) and a schematic illustration of solvent-induced compaction leading to void formation.

**Figure 3:**
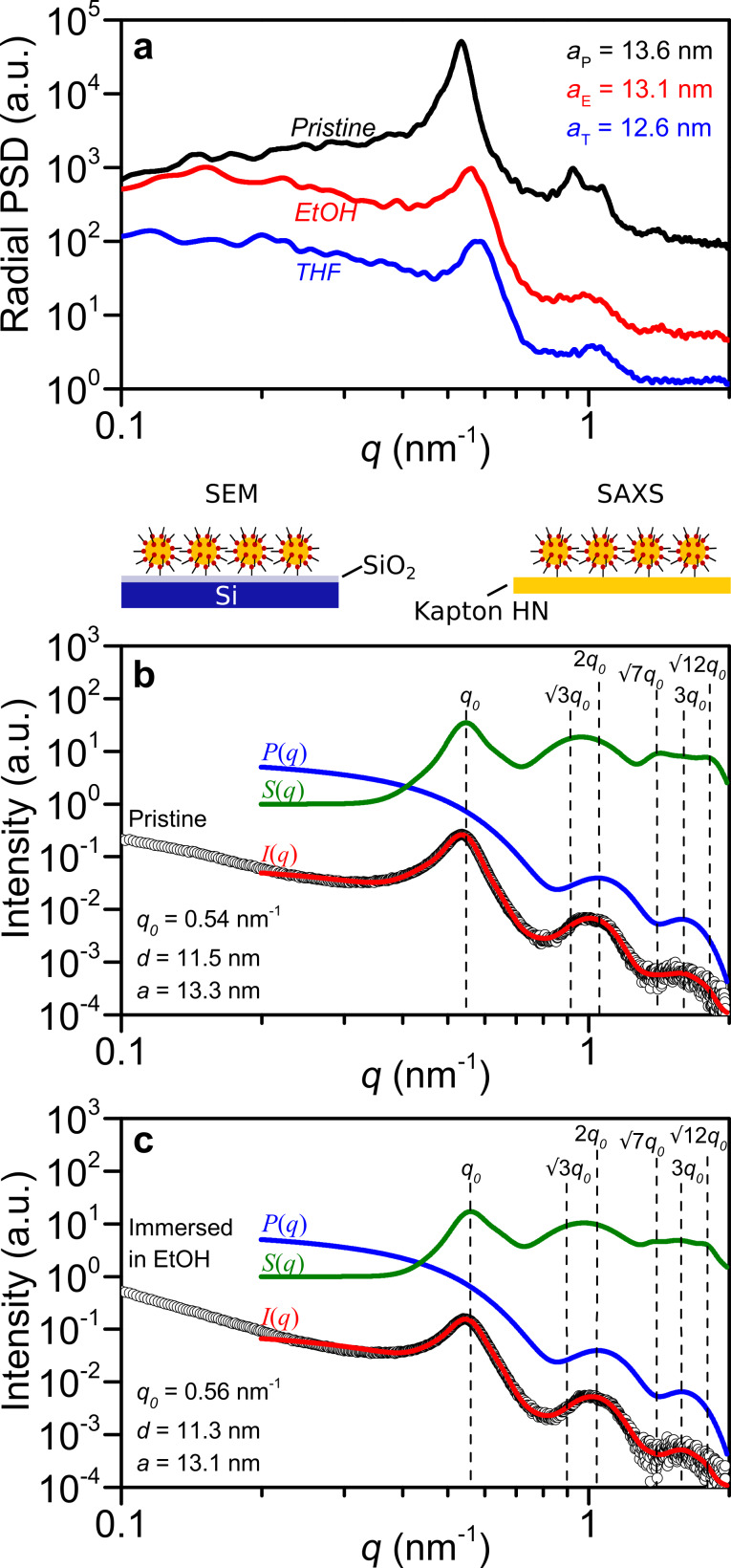
Evolution of gold nanoparticle monolayers upon solvent immersion measured by SEM and SAXS. (a) Radial power spectral density calculated from the respective SEM images in Figure 2 and the corresponding extracted lattice parameters. SAXS intensity profiles are shown for (b) a pristine nanoparticle monolayer and for (c) a nanoparticle monolayer immersed in EtOH. Fitting curve: *I*(*q*) = *NP*(*q*)*S*(*q*) + bkg. Form factor: *P*(*q*) = polydisperse spherical nanoparticle model. Structure factor: *S*(*q*) = 2D hexagonal packing of spheres.

Figure 2a shows an SEM image of a pristine gold nanoparticle monolayer. Nanoparticles are hexagonally ordered within grain boundaries. Small voids between grains result from the self-assembly process. We masked voids in five SEM images at different sample locations, neglecting voids smaller than 25 nm^2^, as an upper limit for tolerances in interparticle distance variations. On average, these voids occupy 5% of the total area of pristine monolayers. Larger voids evolve in nanoparticle monolayers upon immersion in EtOH or THF as demonstrated by the SEM images in Figure 2b,c. We measure an average void density of 19% and 22% after EtOH and THF immersion, respectively. For clarity, masked SEM images for void area measurements are included in the Supporting Information File 1 (Figures S1–S3). The evolution of the void distribution upon immersion is shown in Figure 2d. The average number of nanoparticles per area for the pristine monolayer, measured at five different locations, varies by 1%. On average, after EtOH and THF immersion we measure 1.6% less and 2.5% more nanoparticles per unit area, respectively. The numbers of particles per area after immersion lie within one standard deviation of the measured number of particles per area on pristine monolayers. Therefore, we exclude nanoparticle removal by solvent immersion as the main reason for void formation. In combination with the measured increase in conductance these observations hint at a decreasing average particle separation upon immersion as illustrated in the schematic in Figure 2d. In Figure 3a we plot the radial power spectral density of the SEM images shown in Figure 2. The extracted lattice constant for the pristine nanoparticles is 13.6 nm decreasing to 13.1 nm and 12.6 nm after immersion in EtOH and THF, respectively. For comparison, in an alkane chain three carbon atoms are separated by 0.25 nm. As an estimation for the compaction of the monolayer, a reduction in lattice constant of a hexagonal unit cell by 1 nm would induce a 14% decrease in the occupied surface.

The results from the radial power spectral density distribution are confirmed by SAXS measurements of gold nanoparticle monolayers deposited on a pristine Kapton HN substrate, demonstrating that the observed decrease in particle separation is not linked to the substrate. Figure 3b,c show the measured and calculated intensity profiles before and after immersion in EtOH, respectively. Firstly, the form factor, *P*(*q*) – blue curve in Figure 3b,c – of the gold nanoparticle was obtained by fitting the intensity profile of dilute dispersions (0.1 and 0.01 wt % in water) with the polydisperse spherical nanoparticle model (Supporting Information File 1, Figures S4, and S5). This allowed us to evaluate the radius of the particles either from the fitting model (*r* = 5.32 nm, *p* = σ·*r*^−1^ = 0.09) or from the analysis in the Guinier regime (*R*_g_ = 4.46 nm and *R* = 5.76 nm) (Supporting Information File 1, Figure S6). Secondly, the intensity profile for the pristine nanoparticle monolayer and for the nanoparticle monolayer immersed in EtOH was fitted – red curve in Figure 3b,c – by taking into account the form factor *P*(*q*) of the dispersed gold nanoparticles in water – blue curve – and the structure factor *S*(*q*) of the 2D version of a close-packing lattice system with paracrystalline distortion (also called lattice factor, *Z*(*q*)) – green curve [20]. Finally, from the evaluation of the first peak, *q*_1_, from the structure factor of a 2D hexagonal packing, the lattice parameter, *a*, was evaluated from the relation *a* = 2π/(*q*_1_cos 30°). The lattice parameter obtained by SAXS evaluation matches the SEM measurement for the EtOH immersed sample and deviates for the pristine monolayer by 2.2%.

**Figure 4:**
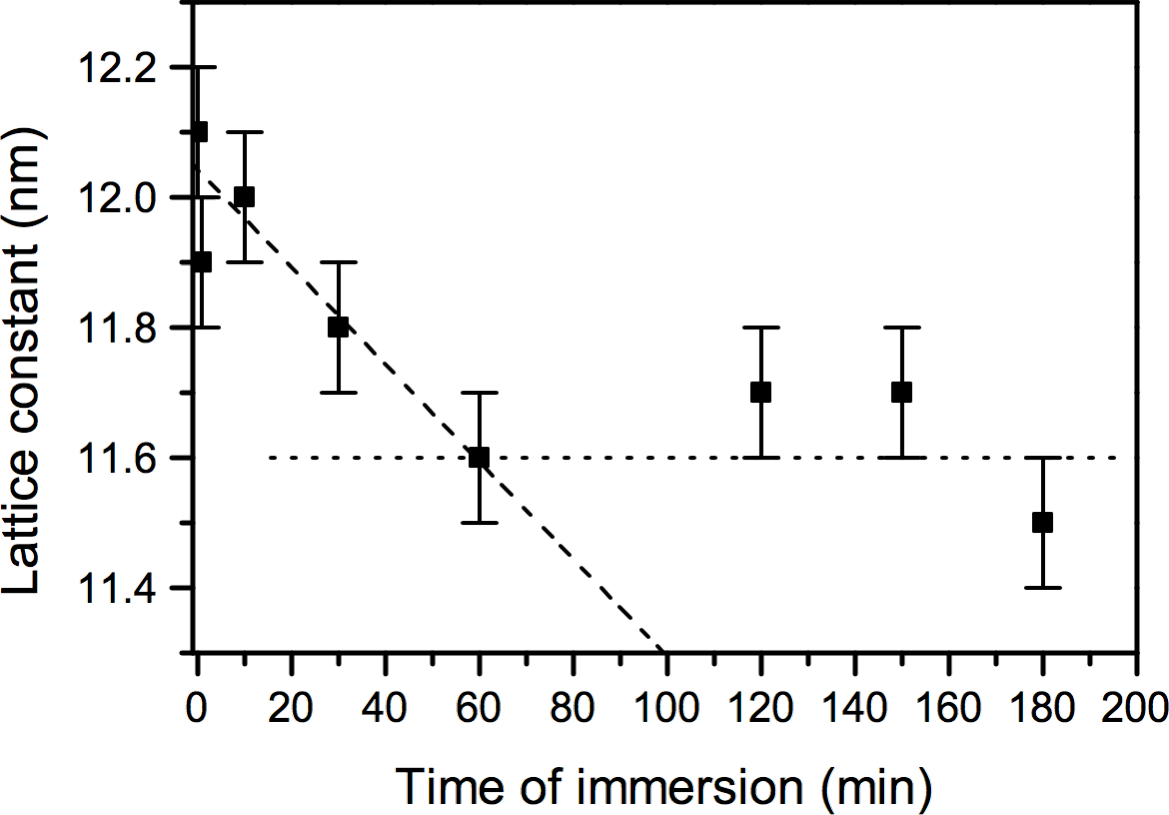
The change in lattice constant extracted from the radial power spectral density of SEM images taken from monolayers immersed for different durations. Dashed and dotted lines indicate trends for different durations.

A major question relates to the cause of the observed compaction and, therefore, the lateral relocation of particles on the surface. To this end, we studied the time scale on which compaction occurs. The evolution of the lattice constant as a function of immersion duration is shown for EtOH in Figure 4 for 12.3 nm gold nanoparticles (hydrodynamic diameter measured by dynamic light scattering).

We extracted the lattice constants from radial power spectral density profiles of SEM images. The lattice constant of a pristine sample coincides with the lattice constant of a monolayer immersed for 10 s. From Figure 4, we can conclude that the compaction process is completed within one hour. This excludes that capillary forces originating from a drying solvent meniscus cause the compaction of the monolayer, because it would be independent of the immersion duration. The observed time dependence can be expected if the decrease in lattice constant is driven by an energy minimization process. Attractive van-der-Waals forces between adjacent nanoparticles can cause agglomeration of nanoparticles, which we observed after removing the alkane ligands by UV/ozone treatment. However, van-der-Waals forces rather decrease slightly in a liquid medium in comparison to air.

A slow compaction of the nanoparticle monolayer as we observed may be caused either by a collapse of alkyl tails or by partial interdigitation of alkyl tails between nanoparticles. The good solubility of 1-dodecanethiol in EtOH and THF rather points towards interdigitation than a collapse of alkyl tails. Badia et al. already suggested interdigitation of alkanes surrounding gold nanoparticles and found a temperature dependence on the alkyl chain ordering by transmission electron microscopy [21]. On flat surfaces alkane chains are known to form interdigitated layers revealed by scanning tunneling microscopy [22–23]. Interestingly, the measured decreases in lattice constant after solvent immersion correspond to two and four times the distance between three carbon atoms in an alkyl chain.

The conductance, *G*, of a metal–molecule–metal junction increases exponentially with decreasing interparticle distance, *d*, i.e., *G* 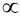 exp(−β*d*) [24–28]. This relation was shown to be valid for single junctions in alkanethiolated nanoparticle networks [5,29]. Dodecanethiol cannot bridge two gold nanoparticles. However, considering the gap size and the length of dodecanethiol, tails of molecules linked to opposing nanoparticles overlap. For the following calculation of conductance increase we neglect the influence of percolation of charge carriers on the overall conductance and assume a hexagonal lattice. In this case, the total conductance of the lattice is directly proportional to the conductance of a single junction [3]. The estimated conductance change originating from the measured change in lattice constant, Δ*a*, follows then the relation *G*′/*G* = exp(βΔ*a*). The decay constant, β, was found to be between 1.05 Å^−1^ and 0.76 Å^−1^ for alkane chains with one and two chemisorbed contacts [30]. Since Δ*a* = 0.5 nm for immersion in EtOH, the conductance would increase by a factor between 190 and 45. On average, the conductance of our monolayers increased by 36 during EtOH immersion, which is slightly lower than the calculated values. As mentioned above, the total conductance in a nanoparticle network also depends on the number of percolation paths [11–12]. If the decrease in lattice constant leads to the formation of large voids and cracks in the nanoparticle monolayer, the amount of good electric connections decreases. Therefore, we measure lower conductance than estimated by just considering the decrease in lattice constant. This effect is much more pronounced for immersion in THF since more large area voids are formed as can be seen from the histograms in Figure 2d. Following the same calculation as above for THF immersion the conductance would increase by a factor ranging from 2000 to 36000, but we only measured an average conductance increase by a factor of 22.

**Figure 5:**
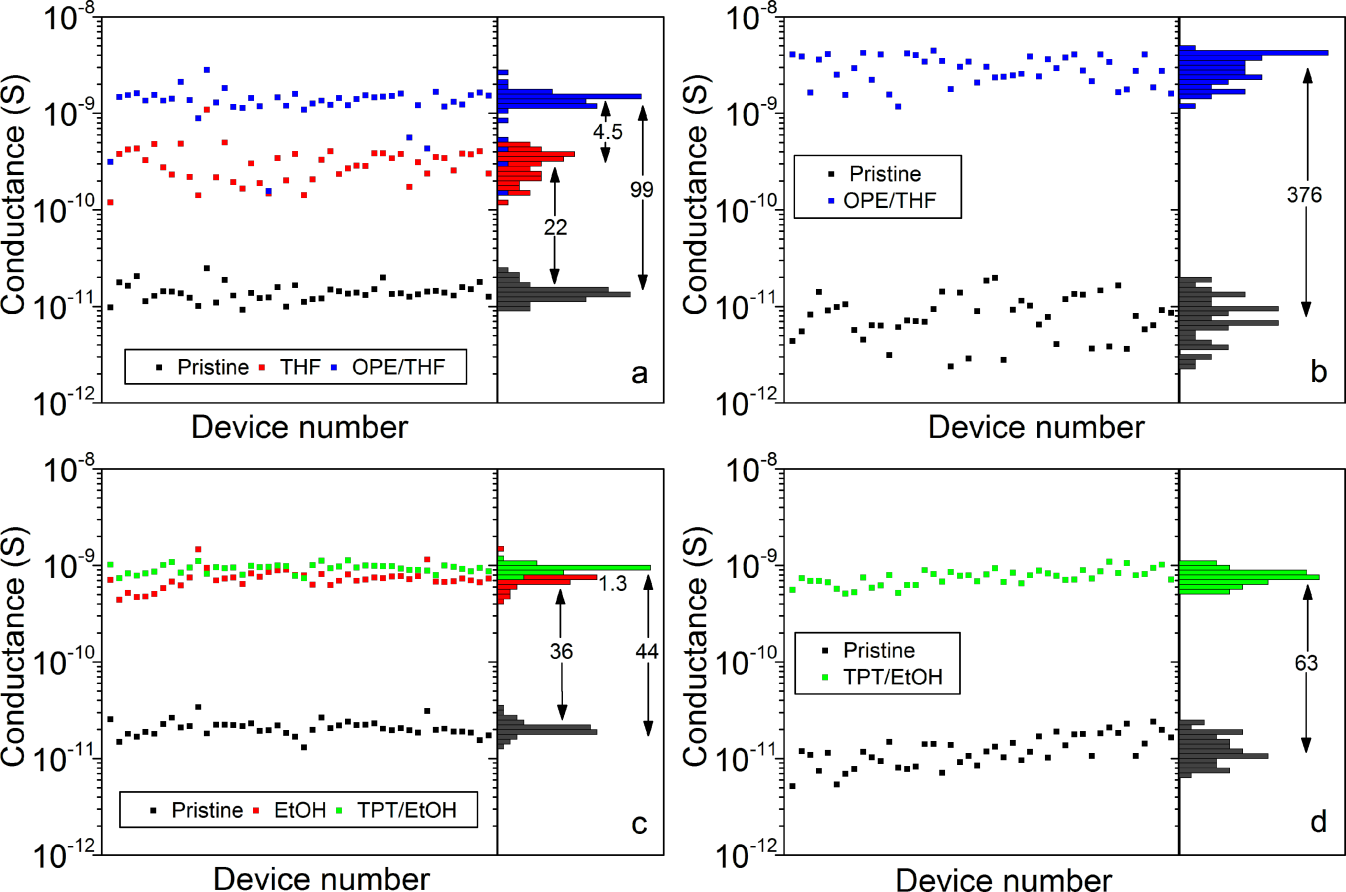
Influence of solvent alone and of solvent containing conjugated ligands on device conductance. (a) Pristine devices, immersed in THF, and immersed in THF with OPE. (b) Pristine devices, and immersed in THF with OPE. (c) Pristine devices, immersed in EtOH, and immersed in EtOH with TPT. (d) Pristine devices, and immersed in EtOH with TPT. Devices for each graph were fabricated on different chips. Histograms of device conductance and the relative conductance increase are shown on the right side of each plot.

In literature changes in conductance of nanoparticle monolayers after immersion in a solvent containing more conductive molecules are interpreted as an exchange of molecular ligands [3,5–6]. Our data showing a conductance increase by immersion in a pure solvent alone raise the question to what extent an exchange with more conductive molecules contributes to the overall increase in conductance. To this end, we immersed the samples for 20 h in a solvent containing molecules of higher conductivity. Dithiolated oligo(phenylenethynylene) (OPE) was dissolved in THF and *p*-terphenylthiol (TPT) was dissolved in EtOH. We compare the conductance of devices which were first immersed in pure solvent (Figure 5a,c) with devices which were directly immersed in the molecule containing solvent (Figure 5b,d). The average conductance of devices that were first immersed in pure THF further increases by a factor of 4.5 after immersion in OPE/THF (Figure 5a). Pristine devices directly immersed in THF with OPE exhibit a 3.8 times higher relative conductance increase compared to sequential immersion (Figure 5b).

The measured conductance values after direct OPE/THF immersion are in agreement with previous experiments [3]. In both cases for OPE/THF immersion the conductance does not increase as much as one would expect from the decrease in lattice constant alone. Comparing the conductance values in Figure 5a,b after OPE/THF immersion with the conductance of OPE-dithiol measured by mechanical break junction experiments (9.3 × 10^−9^ S) [31], one might infer a high yield of exchanged molecules. However, the average conductance of the THF immersed devices (red data points in Figure 5a) already reaches 3 × 10^−10^ S, even though dodecanethiol is 100 times less conductive than OPE-dithiol [25,31].

The need to consider compaction of the nanoparticle monolayer upon solvent immersion is even more urgent in the case of TPT. We chose the TPT-monothiol to ensure that molecules do not link pairs of gold nanoparticles. Nevertheless, an increase in conductance upon exchange of alkanethiols with TPT may be expected due to π–π stacking [32]. The immersion in TPT/EtOH following immersion in pure EtOH (Figure 5c) results in almost no increase in device conductance (factor 1.3). Also direct immersion of pristine devices in TPT/EtOH does not result in much higher device conductance compared to immersion in pure EtOH as shown in Figure 5d. The relative conductance increase for devices directly immersed in TPT/EtOH compared to devices first immersed in pure EtOH is 1.4 times higher. In the case of TPT/EtOH immersion, the conductance increase is dominated by the compaction of the nanoparticle monolayer upon immersion. We extracted the lattice constant 13.3 nm and 12.5 nm from the radial power spectral density (data not shown) of SEM images of the nanoparticle monolayers after TPT/EtOH and OPE/THF immersion, respectively. Therefore we exclude that molecules in the solvent inhibit compaction of the monolayer during immersion.

## Conclusion

Considering all aspects we attribute a major role to the solvent in contributing to the observed conductance increase of nanoparticle monolayers undergoing liquid phase molecular exchange protocols. Solvent induced decreases in lattice constant need to be taken into account for comprehensive interpretation of the electronic measurements on nanoparticle monolayers. We showed here that self-assembly of nanoparticle monolayers on liquid phase followed by micro-contact printing leads to a morphology that is able to further evolve upon exposure to another solvent. This simple effect not only allows further compaction of supported nanoparticle monolayers but could equally well find application in sensing.

## Experimental

### Sample preparation

Gold nanoparticles were synthesized in deionized water by reducing tetrachloroauric acid with trisodium citrate and tannic acid [14]. The synthesis resulted in a nanoparticle concentration of 10^13^ NP/mL. The nanoparticle hydrodynamic size and polydispersity was measured by dynamic light scattering with a Malvern Zetasizer. A hydrodynamic diameter of 12.3 nm was measured for gold nanoparticles used for immersion duration study and 13.4 nm for all other experiments. 10 mL of the aqueous colloid were centrifuged (13000 rpm) for 30 min and redispersed in ethanol. 200 µL 1-dodecanthiol were added to the colloidal dispersion. The nanoparticles fully precipitated after 48 h and the supernatant ethanol was removed with a pipette. A subsequent washing step with ethanol was performed to dispose unbound alkanethiols. The precipitated nanoparticles were dispersed in 4 mL of chloroform and sonicated for 10 min [15]. 400 µL of this dispersion were deposited on a convex water surface inside a poly(tetrafluoroethylene) (PTFE) ring supported by glass slides in a petri dish [16]. After chloroform evaporation (10 min) the self-assembled nanoparticle monolayer was picked up by a structured PDMS (Sylgard 184, Corning) stamp with 10 µm deep grooves. The Si/SiO_2_ substrate was rinsed with acetone and isopropyl alcohol, dried with a stream of nitrogen and placed on the PDMS stamp for 10 s [17].

### Immersion

All immersion experiments were conducted under nitrogen atmosphere for 20 h. The solvents were bubbled for 2 min with nitrogen prior to immersion. For OPE/THF and TPT/EtOH immersion the molecular concentration was 1 mM. In the case of OPE, the acetyl protecting group was removed by addition of 20 µL ammonia (30%) in 3 mL solution while bubbling with nitrogen. The samples were immersed upside down. After immersion the samples were rinsed with THF or EtOH and dried under nitrogen flow [3].

### Contact deposition

Electric contacts were applied by shadow mask evaporation. We aligned a 400 mesh TEM grid with the printed lines on the substrate and deposited 3 nm titanium and 65 nm gold by electron-beam evaporation at a pressure below 1 × 10^−6^ mbar.

### Device characterization

We acquired *I*–*V* curves in a two-probe setup on a Signatone probe station using a Keithley 6517A electrometer and a Stanford Research Systems SIM928 voltage source.

### SAXS measurements

The SAXS experiments were performed using a Rigaku MicroMax-002+ microfocused beam (40 W, 45 kV, 0.88 mA). Cu Kα radiation (λ_CuKα_ = 1.5418 Å) was collimated by three pinhole collimators (0.4, 0.3, and 0.8 mm). The scattered X-ray intensity was detected by a two-dimensional Triton-200 gas-filled X-ray detector (20 cm diameter, 200 μm spatial resolution) covering a momentum transfer range of 0.1 nm^−1^ < *q* < 2 nm^−1^, where *q* = 4π sin θ/λ_CuKα_, and 2θ is the scattering angle. The scattering intensity profiles were analyzed using the software SANS & USANS Analysis with IGOR Pro and OriginPro 10 [33]. Information about the measurement of nanoparticle size and distribution with SAXS is provided in Supporting Information File 1.

## Supporting Information

File 1 Masking of SEM images and additional experiments.
