# Supplementary material for: Solvent-mediated conductance increase of dodecanethiol-stabilized gold nanoparticle monolayers
Source: Beilstein J Nanotechnol. 2016 Dec 23;7:2057–64. doi: 10.3762/bjnano.7.196 (PMC5238686; doi:10.3762/bjnano.7.196)
Supplement: File 1 — Masking of SEM images and additional experiments. [file Beilstein_J_Nanotechnol-07-2057-s001.pdf]

Supporting Information

for

Solvent-mediated conductance increase of dodecanethiol-stabilized gold nanoparticle monolayers

Patrick A. Reissner[1]*, Jean-Nicolas Tisserant[1], Antoni Sánchez-Ferrer[2], Raffaele Mezzenga[2] and Andreas Stemmer[1]*

Address: [1]ETH Zürich, Nanotechnology Group, Säumerstrasse 4, CH-8803 Rüschlikon, Switzerland and [2]ETH Zürich, Laboratory of Food and Soft Materials, Schmelzbergstrasse 9, CH-8092 Zürich, Switzerland

Email: Patrick A. Reissner - preissner@ethz.ch; Andreas Stemmer - astemmer@ethz.ch

* Corresponding author

Masking of SEM images and additional experiments

Contents

I. Masks for void area measurement

II. Size and distribution measurement of gold nanoparticles

I. Masks for void area measurements

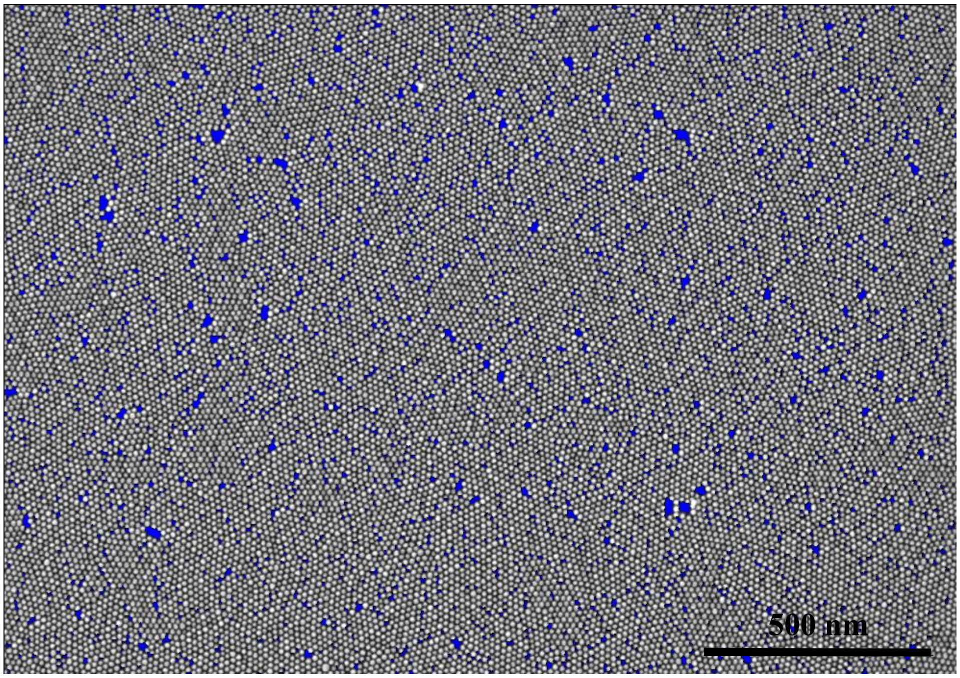

Figure S1: SEM image of a pristine gold nanoparticle monolayer. Voids larger than 25 nm^2 are marked in blue.

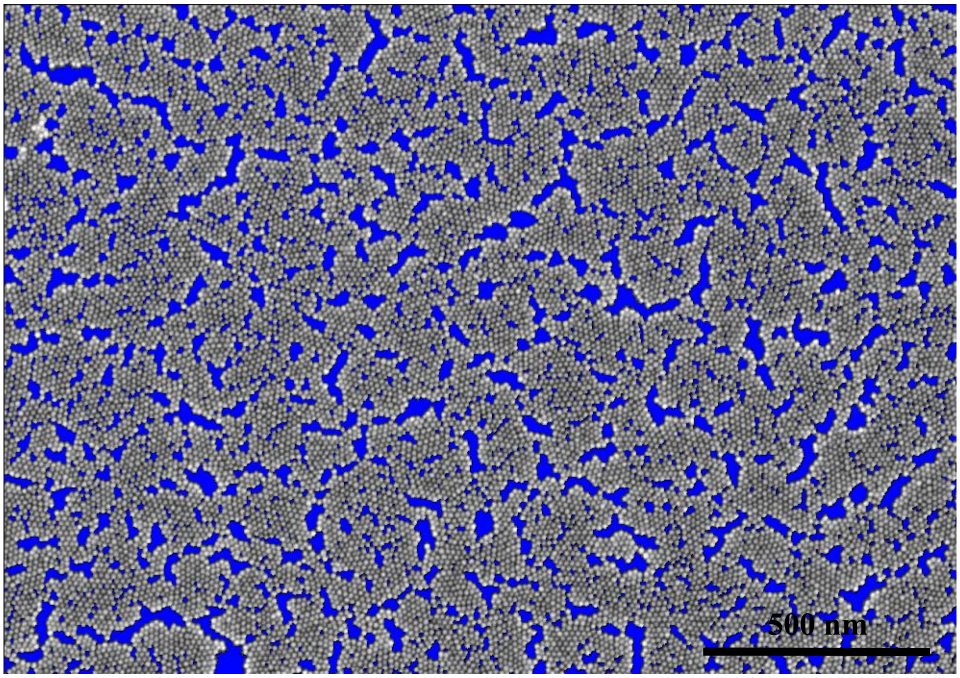

Figure S2: SEM image of a gold nanoparticle monolayer after immersion in ethanol. Voids larger than 25 nm^2 are marked in blue.

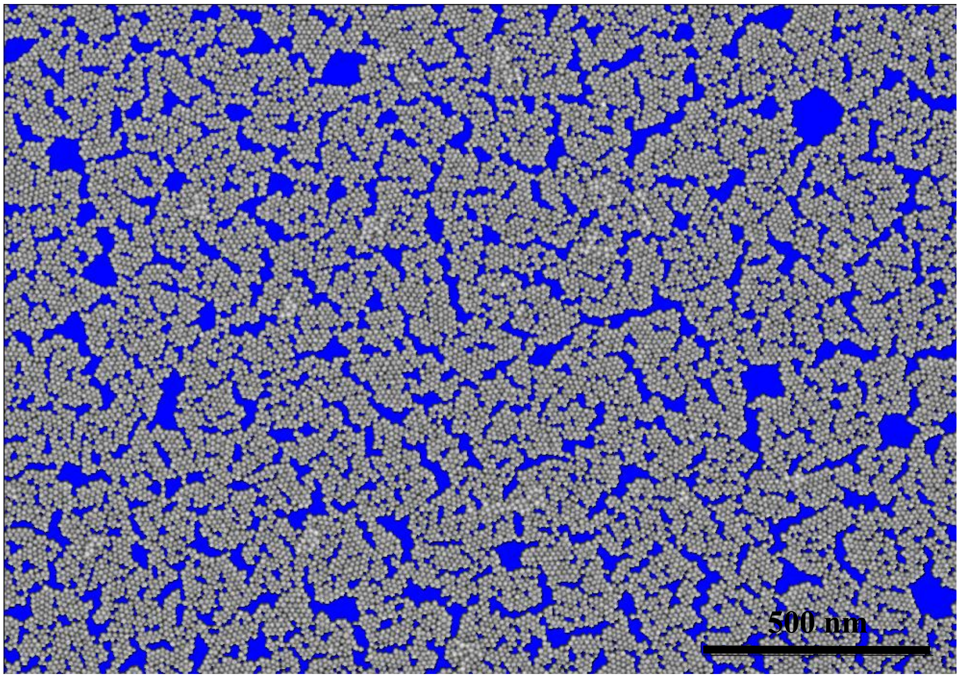

Figure S3: SEM image of a gold nanoparticle monolayer after immersion in tetrahydrofuran. Voids larger than 25 nm^2 are marked in blue.

II. Size and distribution measurement of gold nanoparticles

In order to get information about the size and distribution of the spherical gold nanoparticles, small-angle X-ray scattering (SAXS) experiments were performed on aqueous dispersions of gold nanoparticles at two different concentrations (0.1 and 0.01 wt %). First, all scattering data were dark current and transmission corrected, and the corrected capillary signal was subtracted (Figure S4). Second, the concentration factor and the solvent subtraction were conducted for all samples in order to verify that all scattering profiles fell on the same master curve (Figure S5), indicating that the concentrations were sufficiently diluted to leave only the form factor $P(q) \sim I(q)/c$ visible in the curves, with the structure factor $S(q)$ independent from concentration and $S(q)$ = 1. Then, the form factor $P(q)$ was calculated from the corresponding scattering pattern of the highest concentration by applying a spherical polydisperse particle model, and estimating the radius of the particle (r = 5.32 ± 0.05 nm) and its polydispersity ($p = \sigma \cdot r^{-1}$ = 0.09 ± 0.01). The scattering length densities used for the gold nanoparticles and the

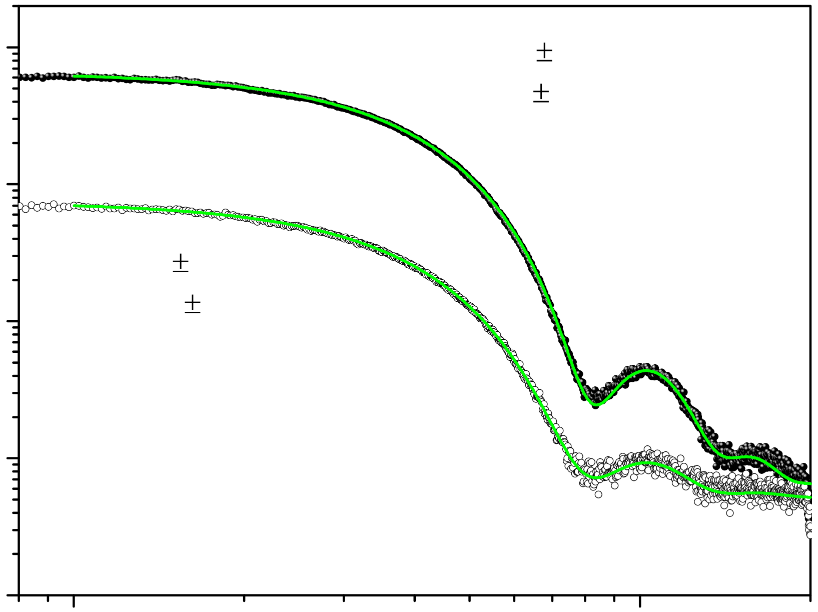

Figure S4: 1D SAXS intensity profile for the 0.1 and 0.01 wt-% Au NPs in water and the corresponding polydisperse spherical model fitting curves.

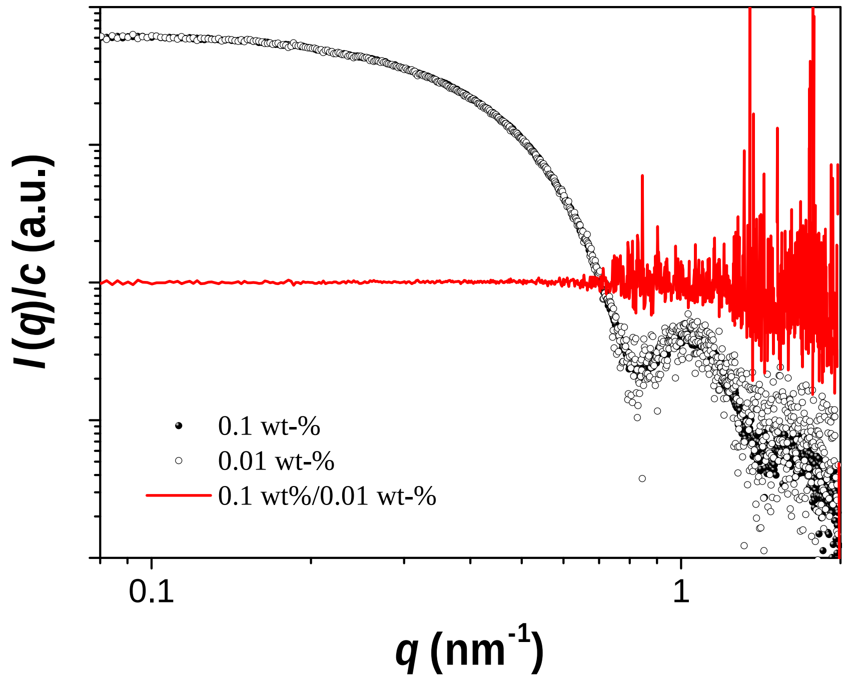

Figure S5: Normalized 1D SAXS intensity profile for the 0.1 and 0.01 wt-% Au NPs in water, showing that the intensity profile corresponds to the spherical form factor.

solvent (water) were $\rho_{Au} = 1.3286{\cdot}10^{-2}$ nm^{-2} and $\rho_{water} = 9.5096{\cdot}10^{-4}$ nm^{-2}, respectively. Guinier analysis on this nanoparticle system leads towards values for the radius of gyration and nanoparticle radius of 4.459 ± 0.001 nm and 5.757 ± 0.002 nm, respectively (Figure S6). Finally, the scattering intensity profile of the gold nanoparticles monolayers deposited on Kapton were measured, and fitted taking into account the form factor P(q) of the dispersed gold nanoparticles in water and the structure factor S(q) of the 2D version of cubic lattice system with paracrystalline distortion (also called lattice factor, Z(q)) (Figure 3b and 3c in the main manuscript).

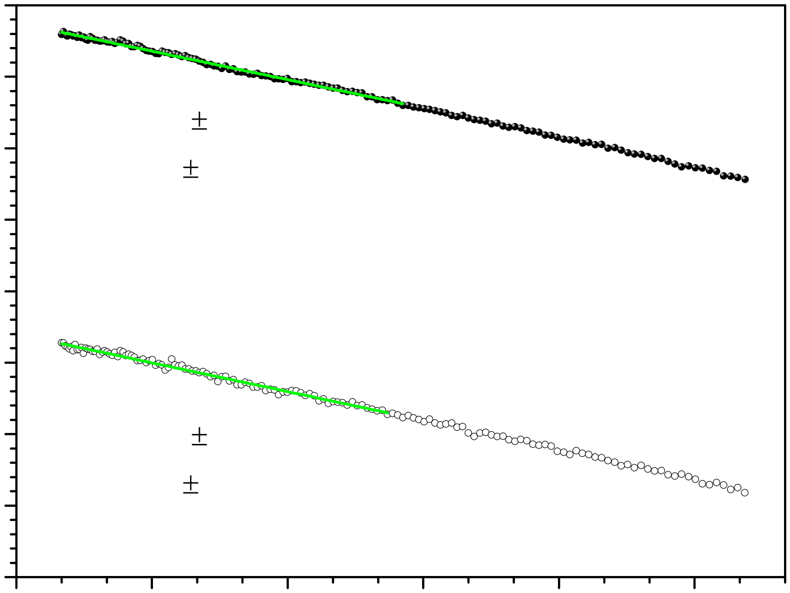

Figure S6: Guinier analysis for the 0.1 and 0.01 wt-% Au NPs in water and the corresponding radius of gyration and radius of the particles.
